# Overexpression of an Osa-miR162a Derivative in Rice Confers Cross-Kingdom RNA Interference-Mediated Brown Planthopper Resistance without Perturbing Host Development

**DOI:** 10.3390/ijms222312652

**Published:** 2021-11-23

**Authors:** Wenzhong Shen, Shanni Cao, Jinhui Liu, Wenqing Zhang, Jie Chen, Jian-Feng Li

**Affiliations:** 1 State Key Laboratory of Biocontrol, Guangdong Provincial Key Laboratory of Plant Resources, School of Life Sciences, Sun Yat-sen University, Guangzhou 510275, China; shenwzh5@mail.sysu.edu.cn (W.S.); caoshn@mail2.sysu.edu.cn (S.C.); liujh89@mail2.sysu.edu.cn (J.L.); lsszwq@mail.sysu.edu.cn (W.Z.); 2 Guangdong Provincial Key Laboratory of High Technology for Plant Protection, Plant Protection Research Institute, Guangdong Academy of Agricultural Sciences, Guangzhou 510640, China

**Keywords:** brown planthopper, cross-kingdom RNA interference, *NlTOR*, osa-miR162a, rice

## Abstract

Rice is a main food crop for more than half of the global population. The brown planthopper (BPH, *Nilaparvata lugens*) is one of the most destructive insect pests of rice. Currently, repeated overuse of chemical insecticides represents a common practice in agriculture for BPH control, which can induce insect tolerance and provoke environmental concerns. This situation calls for innovative and widely applicable strategies for rice protection against BPH. Here we report that the rice osa-miR162a can mediate cross-kingdom RNA interference (RNAi) by targeting the *NlTOR* (*Target of rapamycin*) gene of BPH that regulates the reproduction process. Through artificial diet or injection, osa-miR162a mimics repressed the *NlTOR* expression and impaired the oviposition of BPH adults. Consistently, overproduced osa-miR162a in transgenic rice plants compromised the fecundity of BPH adults fed with these plants, but meanwhile perturbed root and grain development. To circumvent this issue, we generated osa-miR162a-m1, a sequence-optimized osa-miR162a, by decreasing base complementarity to rice endogenous target genes while increasing base complementarity to *NlTOR*. Transgenic overexpression of osa-miR162a-m1 conferred rice resistance to BPH without detectable developmental penalty. This work reveals the first cross-kingdom RNAi mechanism in rice-BPH interactions and inspires a potentially useful approach for improving rice resistance to BPH. We also introduce an effective strategy to uncouple unwanted host developmental perturbation from desirable cross-kingdom RNAi benefits for overexpressed plant miRNAs.

## 1. Introduction

Rice (*Oryza sativa*) is one of the most important staple food crops worldwide. The brown planthopper (BPH, *Nilaparvata lugens*) is an infamous rice-specific pest that has plagued numerous times in history and caused enormous losses of rice production [1]. In the past years, the application of excessive chemical insecticides remains a prevailing strategy for controlling this pest, at the expense of environmental damage and induced insecticide adaptation of the pests [2]. Another valuable and widely adoptable pest control strategy is the use of transgenic crops that produce the insecticidal Cry toxins from *Bacillus thuringiensis* (Bt) [3]. Unfortunately, Bt crops turned out to be ineffective for controlling piercing-sucking insects, such as BPH, which have developed a high-level resistance to a wide range of traditional pesticides [4,5]. Therefore, novel insecticidal transgene resources urgently need to be explored for rice protection against BPH.

MicroRNAs (miRNAs) have been demonstrated to play vital roles in regulating the development and metamorphosis of insects [6,7,8,9,10]. In BPH, disruption of *Dicer-1*, which encodes a key enzyme in miRNA biosynthesis, substantially impaired the ovarian development and fecundity [11], while the miR-4868b of BPH also regulated the fecundity by targeting the *NlGS* (*Glutamate* *synthase*) gene [12]. Moreover, two conserved miRNAs, miR-8-5p and miR-2a-3p, modulated chitin biosynthesis in response to the ecdysterone signaling [13]. In addition, the miR-2703 and miR-173 of BPH were found to regulate the molting metamorphosis by targeting the *NlCHSA* (*Chitin synthase gene A*) or *Ftz-F1* (*Fushi tarazu –F1)* gene, respectively [14,15]. Notably, miRNAs regulating insect development can also come from plants through food intake. For instance, an insect chitinase-targeting miRNA expressed by transgenic tobacco plants arrested the molting process of the insect larva fed with these plants [16]. More interestingly, a phenomenon called the cross-kingdom RNA interference (RNAi) has been observed during plant-insect interactions, wherein certain plant endogenous miRNAs can be ingested when the insects feed on plant food sources. Subsequently, these plant miRNAs pass through the midgut, enter the hemolymph, accumulate in various tissues, and modulate insect gene expression. For example, the mulberry miRNAs were found to be present in the hemolymph and several tissues of silkworms [17], while plant miR162a from pollens affected the caste formation of honeybees by inhibiting the expression of the *Target of rapamycin* (*TOR*) gene [18]. More generally, plant miRNAs have been shown to enter plant-interacting fungi or mammals to induce cross-kingdom RNAi [19,20,21]. Based on these findings, it is tempting to assume that certain rice miRNA might be able to enter BPH to interfere with the development of the pest. Such knowledge may inspire new insecticidal strategies for BPH management.

Recently, the roles of rice miRNAs in regulating BPH resistance have begun to be appreciated. The osa-miR156 of rice has been demonstrated to negatively regulate BPH resistance, as silencing of osa-miR156 enhanced rice resistance to BPH [22]. The osa-miR396 also negatively regulated BPH resistance in rice through the target gene *OsGRF8* (*Growth regulating factor 8*), which positively modulated the BPH-responsive expression of flavanone 3-hydroxylase. Silencing of osa-miR396 increased flavonoid accumulation and rice resistance to BPH [23]. Of note, thus far no rice miRNA has been identified to promote BPH resistance, let alone enter BPH to reduce the fitness of the pest.

The biosynthesis and accumulation of vitellogenin (Vg) in developing oocytes is of great importance for insect reproduction [24,25,26]. The expression of *Vg* is controlled by the TOR signaling pathway [27,28,29,30]. In BPH female adults with silenced *NlTOR* expression, the transcript level of *Vg* was reduced, which was followed by the inhibition of the ovarian development [31,32]. The decrease of *NlTOR* expression also resulted in retarded development of the accessory gland in BPH male adults [33]. Moreover, inhibition of *TOR* expression could compromise the ovarian development in mosquitoes [34].

In this study, we hypothesized and verified that rice osa-miR162a could silence the *NlTOR* expression in BPH via the cross-kingdom RNAi and impair the fecundity of the insects. Overexpression of osa-miR162a led to augmented rice resistance in BPH, but meanwhile influenced root and grain development due to the perturbation of rice endogenous gene expression. To deal with this problem, we overexpressed an optimized osa-miR162a derivative, which harbors increased mismatches to rice endogenous target genes but decreased mismatches to *NlTOR*. These efforts allowed us to retain the BPH resistance in transgenic overexpression plants but relieve the adverse impact of osa-miR162a overexpression on rice development.

## 2. Results

### 2.1. The NlTOR Gene of BPH Is a Target of Rice Osa-miR162a

**Figure 1:**
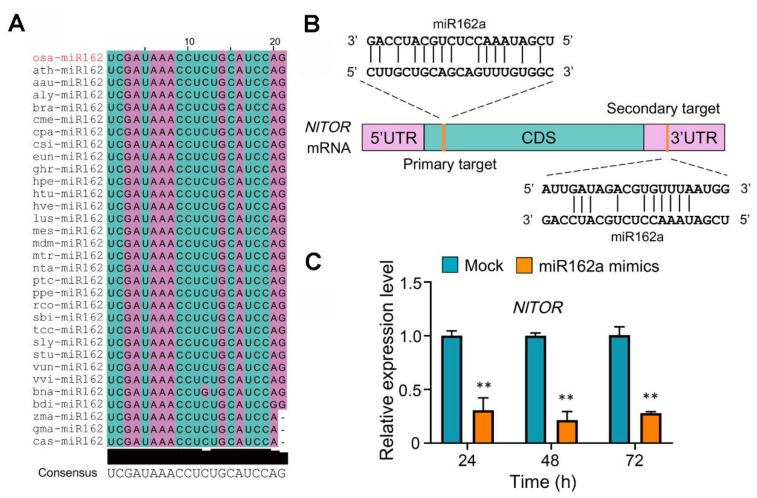
The *NlTOR* gene of BPH is a target of rice osa-miR162a. (**A**) Sequence alignment indicates that miR162 is highly conserved across 32 plant species. The listed plant miR162 sequences were obtained from the miRBase database. (**B**) Putative target sites of osa-miR162 in *NlTOR*. The target sites are indicated by orange sticks. CDS, coding sequence; UTR, untranslated region. (**C**) *NlTOR* expression decreases in BPH female adults after injection with osa-miR162a mimics. The expression levels were quantified by RT-qPCR and the data are presented as mean ± SEM of three biological replicates (*n* = 3). The expression levels of *NlTOR* after injection with negative control miRNA mimics at different time points were normalized as 1. ** *p* < 0.01 (student’s *t*-test).

Recently, the cole crop (*Brassica campestris*) bra-miR162a has been reported to enter the honeybee (*Apis mellifera*) to target and silence the *AmTOR* gene via the cross-kingdom RNAi [18]. As plant miR162 exhibits a remarkable conservation across diverse species of dicots and monocots (Figure 1A) and the insect *TOR* gene also shows a high similarity in sequence and function [28,35], we hypothesized that the *NlTOR* gene of BPH may also be targeted by rice osa-miR162a, which has an identical sequence to bra-miR162a (Figure 1A). In support of this hypothesis, two bioinformatic algorithms, RNAhybrid and miRanda, predicted a primary target site for osa-miR162a in the coding sequence (CDS) of *NlTOR* with 14 bases matching and a secondary target site in the 3′ untranslated region (UTR) with 10 bases matching (Figure 1B).

To quickly evaluate the possibility of cross-kingdom RNAi by rice osa-miR162a in BPH, we examined whether food intake enables osa-miR162a to enter BPH, pass through the midgut, and spread to other tissues. To this end, ten BPH female adults of the first day were fed with artificial diets supplemented with osa-miR162a mimics. After BPH feeding for 24, 48, and 72 h, the presence of osa-miR162a in different tissues was monitored by stem-loop quantitative reverse transcription PCR (RT-qPCR) [36]. The results showed that osa-miR162a could be highly accumulated in several insect tissues, such as midgut, fat body, and hemolymph after 72 h of feeding (Figure S1A). The presence of osa-miR162a inside BPH was further validated by Sanger sequencing of the mixed PCR products from the above tissues (Figure S1B). These results hinted that osa-miR162a is able to pass through the midgut and accumulate in other tissues of BPH through food intake. Next, the BPH female adults of the first day were injected with chemically pure osa-miR162a mimics and the expression level of *NlTOR* was evaluated by RT-qPCR in a time course manner. At 24, 48, and 72 h post injection, the transcript levels of *NlTOR* declined dramatically relative to mock treatment (Figure 1C), suggesting that *NlTOR* can be targeted by osa-miR162a in BPH.

### 2.2. Injected Osa-miR162a Mimics Reduce the Fecundity of BPH

**Figure 2:**
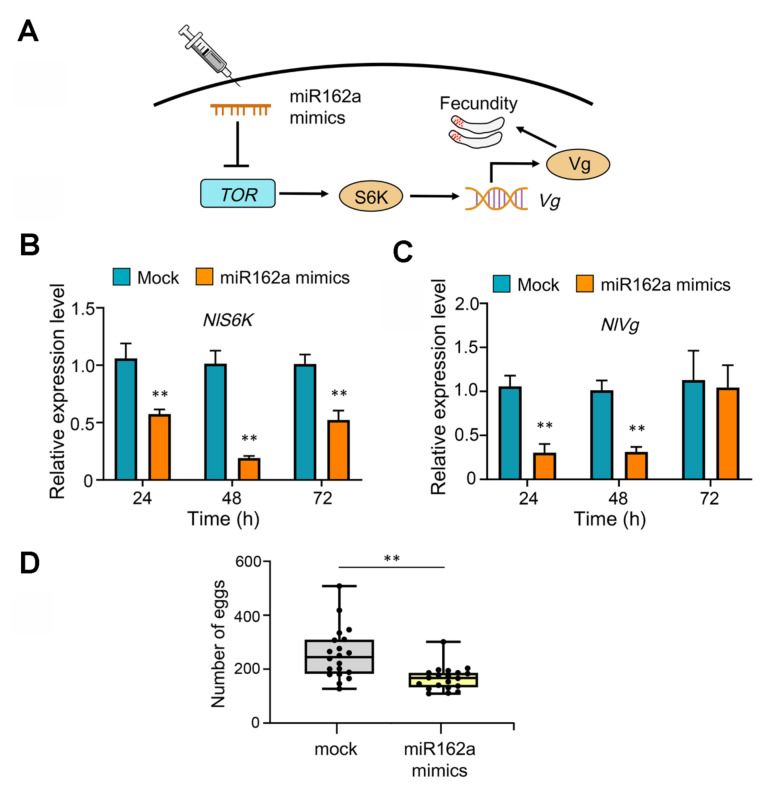
Injected osa-miR162a mimics impair the fecundity of BPH. (**A**) Schematic diagram of the effect of injected osa-miR162a mimics on weakening the TOR signaling-regulated fecundity in BPH. S6K is a downstream player of NlTOR in the TOR signaling and vitellogenin (Vg) is a molecular marker for assessing the fecundity of BPH. (**B**,**C**) Expression of *NlS6K* (**B**) and *NlVg* (**C**) is reduced in female adults injected with osa-miR162a mimics. The expression levels were quantified by RT-qPCR and the data are presented as mean ± SEM of three biological replicates (*n* = 3). The expression levels of *NlS6K* and *NlVg* after injection with negative control miRNA mimics at different time points were normalized as 1. (**D**) Number of eggs per female adult in the fecundity assay. Eggs from 20 female adults were counted and individual data points are shown as dots. ** *p* < 0.01 (student’s *t*-test).

Vg is a frequently used molecular marker for assessing the fecundity of BPH [29], which is regulated by the TOR signaling pathway through the downstream gene *NlS6K* (*Ribosomal S6 kinase 1*) [31] (Figure 2A). The BPH female adults of the first day were injected with osa-miR162a mimics to examine the effects on expression levels of *NlS6K* and *NlVg*. RT-qPCR results showed that the transcript level of *NlS6K* decreased markedly at 24, 48, and 72 h after injection (Figure 2B), while that of *NlVg* declined at 24 and 48 h after injection but was recovered at 72 h (Figure 2C), probably due to compensatory regulation of *Vg* expression by other pathways in BPH under the experimental conditions. Nevertheless, after osa-miR162a mimics were injected into newly emerged female adults, there was a significant reduction in oviposition for insects injected with osa-miR162a mimics relative to those with mock treatment (Figure 2D). Together, these results implied that osa-miR162a can reduce the fecundity of BPH through disturbing the TOR signaling pathway.

### 2.3. Osa-miR162a-m1 Exhibits Minimal Perturbation on Rice Endogenous Gene Expression

**Figure 3:**
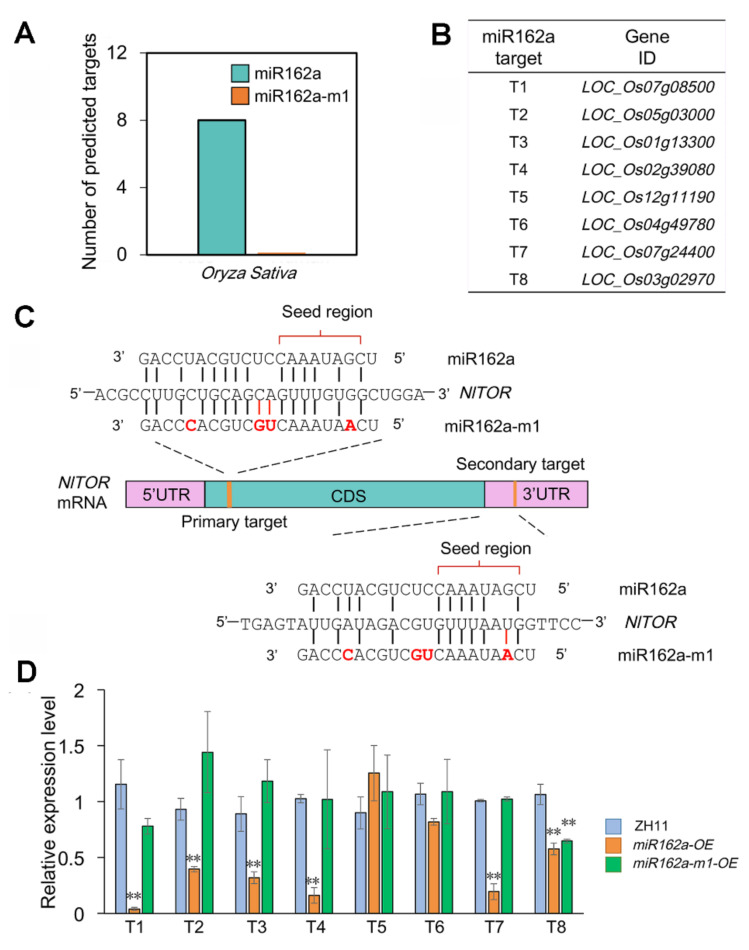
osa-miR162a-m1 exhibits minimal perturbation on rice endogenous gene expression. (**A**) Predicted target gene numbers of osa-miR162a and its derivative osa-miR162a-m1 by WMD. (**B**) Predicted rice target genes of osa-miR162a by WMD. (**C**) osa-miR162a-m1 exhibits additional base matches to the primary target site of *NlTOR* and improved complementarity to the seed region of the secondary target site of *NlTOR* relative to osa-miR162a. The modified nucleotides and newly gained base pairings in osa-miR162a-m1 are highlighted in red. (**D**) Overexpression (OE) of osa-miR162a-m1 exhibits much less perturbation on rice endogenous gene expression than osa-miR162a. The expression levels of putative target genes were determined by RT-qPCR and the data are shown as mean ± SD of three biological replicates (*n* = 3). ** *p* < 0.01 (Student’s *t*-test).

The *NlTOR* gene plays essential roles in governing the development, reproduction, and nutrient sensing of BPH [32,33]. Based on the aforementioned observations, we reasoned that overexpression of osa-miR162a in transgenic rice plants may be able to protect rice from BPH attacks by silencing *NlTOR* via the cross-kingdom RNAi. However, we noted that there are eight rice genes predicted to be targeted by osa-miR162a (Figure 3A,B), suggesting that overexpression of osa-miR162a would affect the expression of these rice genes as well. To overcome this problem, we decided to modify the sequence of osa-miR162a to achieve the following goals: (1) increase its mismatches to putative rice target genes to minimize the silencing of those genes; (2) reduce the mismatches to the *NlTOR* gene to maintain or even strengthen the cross-kingdom RNAi. After screening a number of rationally designed osa-miR162a derivatives using the Web MicroRNA Designer (WMD), a candidate named osa-miR162a modified 1 (osa-miR162a-m1) was selected for two reasons: firstly, no rice gene was predicted to be targeted by osa-miR162a-m1 (Figure 3A); secondly, osa-miR162a-m1 possesses two more base matches to the primary target site of *NlTOR* than osa-miR162a (Figure 3C), thus being potentially more effective in silencing *NlTOR* than the native osa-miR162a.

Next, we generated transgenic rice plants overexpressing osa-miR162a or osa-miR162a-m1 in the ZH11 (Zhonghua 11) background. The accumulation of mature osa-miR162a or osa-miR162a-m1 in the T1 transgenic plants was evaluated using stem-loop RT-qPCR. Multiple osa-miR162a overexpression (*miR162a-OE*) lines and osa-miR162a-m1 overexpression (*miR162a-m1-OE*) lines exhibited readily detectable production of osa-miR162a or osa-miR162a-m1, respectively (Figure S2). The *miR162a-OE* lines 5 and 10 and *miR162a-m1-OE* lines 1 and 3 were selected for further analysis due to their high expression levels and overall wild-type appearance.

To examine the effect of osa-miR162a or osa-miR162a-m1 overexpression on putative target genes in rice, we checked the transcript levels of these genes in the leaf sheaths of the selected transgenic lines. Six of the eight genes, namely *LOC_Os07g08500*, *LOC_Os05g03000*, *LOC_Os01g13300*, *LOC_Os02g39080*, *LOC_Os07g24400*, and *LOC_Os03g02970* (*OsDCL1*, *Dicer-like 1*), were strongly down-regulated in the two *miR162a-OE* lines relative to ZH11 plants (Figure 3D). By contrast, none of the eight genes, except *OsDCL1*, was repressed in *miR162a-m1-OE* lines (Figure 3D). The silencing of *OsDCL1* by osa-miR162a-m1 was completely unexpected, as this gene has not been predicted as a target gene of osa-miR162a-m1 by WMD due to the presence of six mismatches (Figure S3). Nevertheless, the above results suggested that osa-miR162a-m1, upon overexpression, has minimal perturbation on rice endogenous gene expression.

### 2.4. Rice Development Is Not Defected upon Osa-miR162a-m1 Overexpression

**Figure 4:**
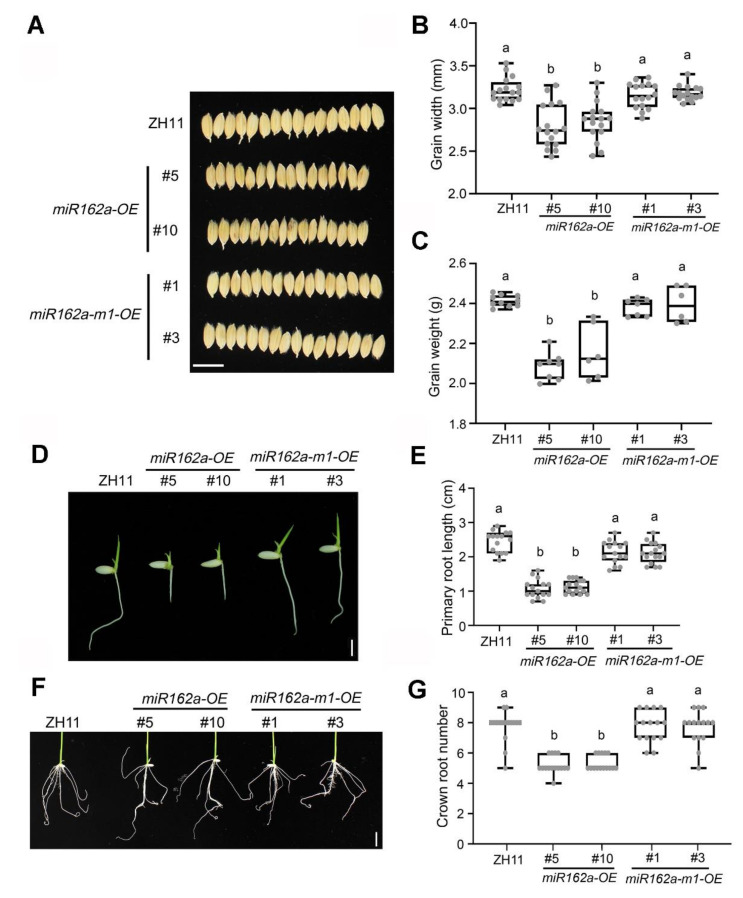
Overexpression of osa-miR162a, but not osa-miR162a-m1, impairs root and grain development in rice. (**A**) Grain development is affected by overexpression (OE) of osa-miR162a, but not osa-miR162a-m1. Scale bar = 1 cm. (**B**) Grain width ranges of 16 randomly selected seeds of indicated genotypes. (**C**) Weight of 100 grains of indicated genotypes. At least six randomly pooled seeds (100 per pool) were evaluated for each genotype. (**D**) Primary root length of three-day-old seedlings is affected by overexpression of osa-miR162a, but not osa-miR162a-m1. Scale bar = 0.5 cm. (**E**) Primary root length quantified using 15 randomly selected seedlings. (**F**) Crown root number of seven-day-old seedlings is affected by overexpression of osa-miR162a, but not osa-miR162a-m1. Scale bar = 0.5 cm. (**G**) Quantification of the crown root number using 15 randomly selected seedlings. In (**B**,**C**,**E**,**G**), measurements are plotted as boxes and whiskers displaying mean ± SD. Individual data points are shown as dots. Significant differences were determined using one-way ANOVA followed by Tukey post hoc test. Letters indicate significantly different groups.

We further assessed the effect of osa-miR162a or osa-miR162a-m1 overexpression on rice growth and development. Although the heights and tiller numbers of one-month-old plants showed no significant difference between ZH11, *miR162a-OE*, and *miR162a-m1-OE* plants (Figure S4A–C), we noted that both the grain widths and grain weights of *miR162a-OE* lines were significantly reduced relative to ZH11 plants (Figure 4A–C). In agreement with these results, a recent study also reported that overproduction of osa-miR162a in transgenic rice plants could generate narrower and lighter grains [37]. Of note, the grain abnormality was not observed in *miR162a-m1-OE* lines (Figure 4A–C). In addition, we noticed that the *miR162a-OE* lines also exhibited compromised root development at the early developmental stage. The three-day-old *miR162a-OE* seedlings had significantly shorter primary roots than ZH11 plantlets (Figure 4D,E), while the seven-day-old *miR162a-OE* seedlings showed slightly reduced crown root numbers relative to ZH11 plantlets (Figure 4F,G). By contrast, there was no obvious difference between *miR162a-m1-OE* lines and ZH11 plants in root development (Figure 4D–G). These results indicated that overexpression of osa-miR162a-m1, unlike osa-miR162a, exerts no adverse effect on rice development.

### 2.5. BPH Adults Fed with miR162a-m1-OE Plants Display Impaired Fecundity

**Figure 5:**
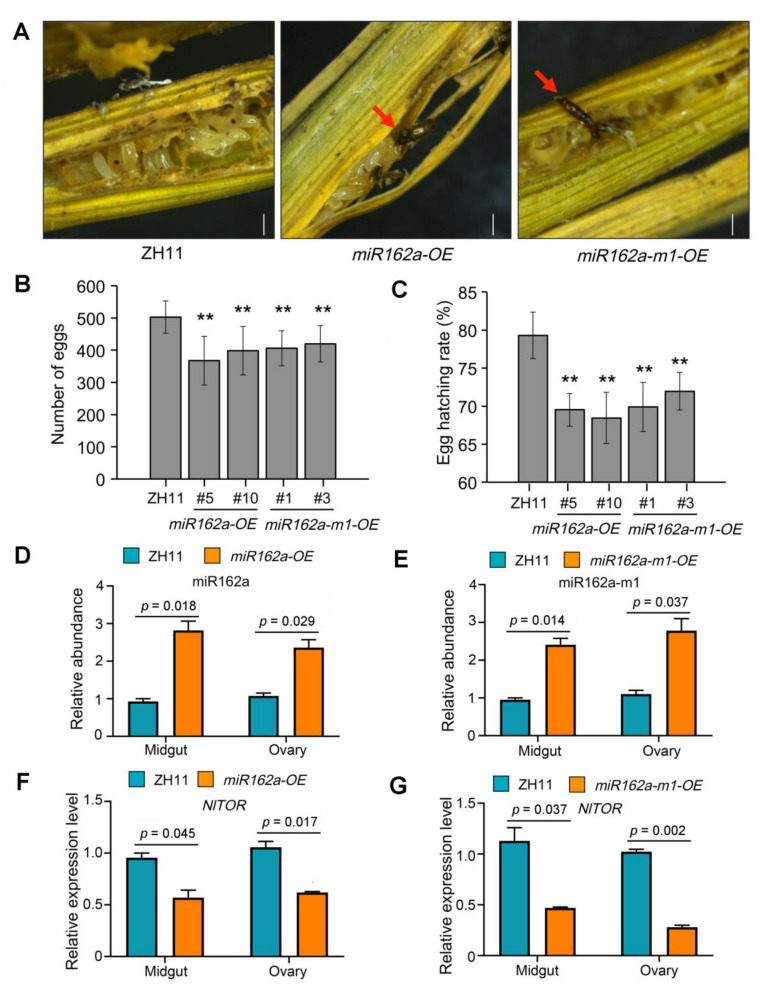
BPH insects fed with *miR162a-OE* or *miR162a-m1-OE* plants show impaired fecundity. (**A**) Insects fed with *miR162a-OE* or *miR162a-m1-OE* plants produce abnormal black eggs or eggs without pigmented eyes. Red arrows indicate black eggs. Scale bar = 0.5 mm. (**B**,**C**) Quantification of egg numbers (**B**) and hatching rates (**C**) for insects fed with indicated genotypes. Eggs from eight female adults were evaluated and the data are presented as mean ± SD. ** *p* < 0.01 (Student’s *t*-test). (**D**,**E**) Increased abundance of osa-miR162a (**D**) or osa-miR162a-m1 (**E**) in the midgut and ovary of insects fed with indicated genotypes. The miRNA abundances were determined by stem-loop RT-qPCR and the data are presented as mean ± SEM. *p* values are shown (Student’s *t*-test). (**F**,**G**) Decreased *NlTOR* expression in the midgut and ovary of insects fed with *miR162a-OE* (**F**) or *miR162a-m1-OE* (**G**) plants. The expression level was determined by RT-qPCR and the data are presented as mean ± SEM. *p* values are shown (Student’s *t*-test). Two biological replicates were conducted and the miRNA or *NlTOR* transcript abundance in insects fed with ZH11 plants was normalized as 1.

As we have shown that injected osa-miR162a mimics could silence the *NlTOR* expression in BPH (Figure 1C) and compromise the oviposition of female insects (Figure 2D), we investigated whether the fecundity of BPH could be affected by continuous feeding on *miR162a-OE* or *miR162a-m1-OE* plants. To this aim, a pair of BPH female adult and male adult were fed with the same rice plant and were mated before oviposition. We found that more black eggs were laid by female adults fed with *miR162a-OE* or *miR162a-m1-OE* plants relative to ZH11 plants (Figure 5A). In addition, many eggs from BPH adults fed with *miR162a-OE* or *miR162a-m1-OE* plants failed to accumulate pigments in the eyes, a sign of abnormal egg development, whereas those from BPH adults fed with ZH11 plants exhibited normally pigmented eyes (Figure 5A). Moreover, the insects fed with *miR162a-OE* or *miR162a-m1-OE* plants produced slightly fewer eggs than those fed with ZH11 plants (Figure 5B). Furthermore, the hatching rates of eggs laid by female adults fed with *miR162a-OE* or *miR162a-m1-OE* plants were significantly lower than those fed with ZH11 plants (Figure 5C).

To examine whether the observed fecundity defects of BPH were associated with the silencing of *NlTOR* via the cross-kingdom RNAi, we interrogated BPH female adults continuously fed with *miR162a-OE* or *miR162a-m1-OE* plants. By stem-loop RT-qPCR, we detected relatively higher abundances of osa-miR162a or osa-miR162a-m1 in the midgut and ovary of female adults fed with *miR162a-OE* or *miR162a-m1-OE* plants than those fed with ZH11 plants (Figure 5D,E). Accordingly, the transcript levels of *NlTOR* in the midgut and ovary of female adults fed with *miR162a-OE* or *miR162a-m1-OE* plants were considerably lower than those fed with ZH11 plants (Figure 5F,G). These results suggested that both osa-miR162a and osa-miR162a-m1 are capable of weakening the fecundity of BPH by silencing *NlTOR* via the cross-kingdom RNAi.

**Figure 6:**
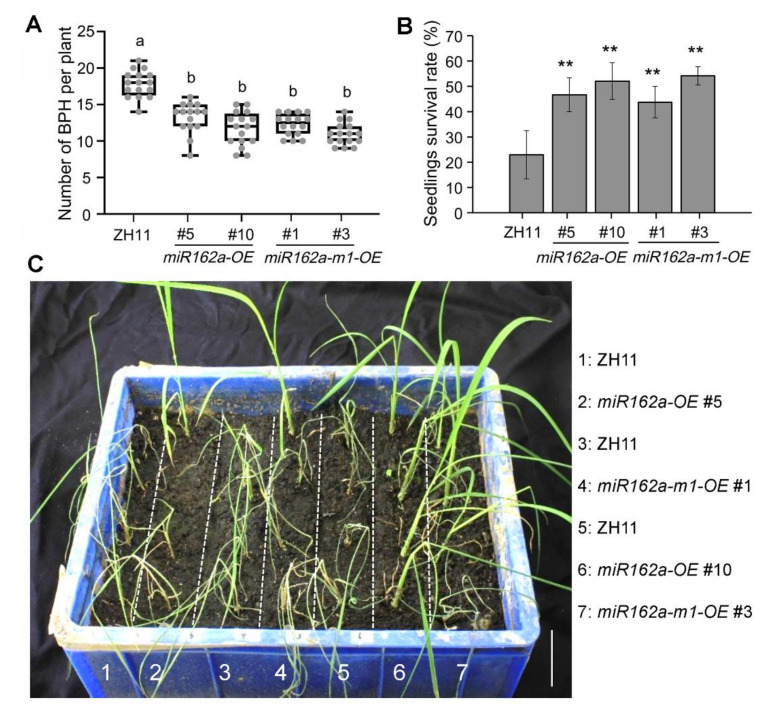
Overexpression of osa-miR162a or osa-miR162a-m1 confers rice resistance to BPH. (**A**) Number of insects settled on indicated genotypes at 48 h after infestation in a host choice test. Fifteen four-leaf-stage young seedlings were infested with 15 nymphs of the third instar per plant. Measurements are plotted as boxes and whiskers displaying mean ± SD. Individual data points are shown as dots. Significant differences were determined using one-way ANOVA followed by Tukey post hoc test. Letters indicate significantly different groups. (**B**) Plant survival rates of indicated genotypes at 10 days after infestation. The data are presented as mean ± SD of three biological replicates (*n* = 3). ** *p* < 0.01 (Student’s *t*-test). (**C**) Representative image of one replicate for (**B**). Scale bar = 4 cm.

Finally, we performed a host choice test between *miR162a-OE*, *miR162a-m1-OE*, and ZH11 plants at the young seedling stage. At 48 h after infestation, fewer insects were found to settle on *miR162a-OE* and *miR162a-m1-OE* plants than on ZH11 plants (Figure 6A). The survival rates of both *miR162a-OE* and *miR162a-m1-OE* seedlings were substantially higher than those of ZH11 plants (Figure 6B,C). These findings indicated that overproduction of osa-miR162a or osa-miR162a-m1 can confer rice resistance to BPH.

## 3. Discussion

In plants, miRNAs act as critical post-transcriptional regulators of gene expression in a broad range of biological processes, including responses to biotic stresses. Many rice miRNAs have been demonstrated to modulate plant immunity against pathogens or insect herbivores by fine-tuning the expression of endogenous target genes [22,23,38,39]. Notably, unlike animal miRNAs, plant miRNAs are 2′-O-methylated at the 3′ end, which is crucial for their increased stability [40]. Some plant miRNAs, as exemplified by miR159 and miR2911, have been reported to be extremely stable even under harsh conditions, such as acidic pH and cooking temperatures [41,42,43]. In the past decade, there has been a growing body of evidence showing that plant miRNAs, partially due to their superb stability, can enter an interacting organism from a different kingdom, such as fungus, insect, or mammal. In that organism, plant miRNAs can mediate the so-called cross-kingdom RNAi to regulate its gene expression and reshape its biological activity, including development, reproduction, and virulence [18,19,20,21]. These groundbreaking discoveries have refreshed our understanding of the functions of plant miRNAs and opened up a new research area in miRNA biology. In this study, we found that intake of rice osa-miR162a mimics by feeding or injection dramatically down-regulated the expression of *NlTOR* in BPH and consequently inhibited the TOR signaling-dictated Vg activity, ultimately leading to impaired fecundity (Figure 1C and Figure 2B–D). Consistently, the BPH adults fed with transgenic rice plants overexpressing osa-miR162a exhibited ovipositing and hatching defects (Figure 5A–C). These results imply that rice osa-miR162a is a previously unknown player for defending BPH via the cross-kingdom RNAi mechanism. Intriguingly, the cole bra-miR162a has also been shown to regulate the caste development in honeybee through the cross-kingdom RNAi [18]. These two studies suggest that the insect *TOR* gene is a conserved target for plant miR162a during plant–insect interactions. It awaits further investigation whether plant miR162a has additional target genes in these insects.

There are two predicted osa-miR162a target sites in the mRNA of *NlTOR*. The primary one is located in the CDS with 14 bases matching, while the secondary one resides in the 3′ UTR with 10 bases matching (Figure 1B). It was thought that animal miRNAs tend to bind the 3′ UTR of the target mRNA, whereas plant miRNAs predominately associate with the CDS [44]. However, more in-depth studies have suggested that both animal and plant miRNAs can bind to the 5′ UTR, 3′ UTR, or CDS of the target transcripts [45,46]. Unlike plant miRNAs, which generally require a perfect or near-perfect complementarity to their target mRNAs, most animal miRNAs exhibit an overall imperfect base pairing with their target mRNAs, except for the “seed region” at the 3′ end of the target sequence. It is also believed that not only the miRNA-target complementarity but also the target accessibility are major determinants for efficient miRNA-target interactions [47,48]. Due to these complexities, we are currently unable to distinguish which predicted target site of *NlTOR* is effectively bound by osa-miR162a inside BPH. Notably, the cole bra-miR162a target site has been demonstrated to be located in the CDS of *AmTOR* in honeybee [18].

The modification of osa-miR162a to osa-miR162a-m1 simultaneously improved the complementarity to the *NlTOR* mRNA at both the primary target site (i.e., from 14 base matches to 16) and the seed region of the secondary target site (i.e., from 6 base matches to 7) (Figure 3C). Therefore, we have anticipated that overexpression of osa-miR162a-m1 would exert a stronger effect than osa-miR162a on weakening the fecundity of BPH. However, we observed comparable reduction in the fecundity of BPH adults fed with transgenic rice plants overexpressing either osa-miR162a or osa-miR162a-m1, suggesting that the complementarity between osa-miR162a and *NlTOR* is not the limiting factor to determine the effectiveness of osa-miR162a-mediated cross-kingdom RNAi.

Although overexpression of osa-miR162a promises an effective and economical means for BPH control in various rice cultivars, the overexpression negatively affected root development and grain production in rice (Figure 4). We found that osa-miR162a could potentially target eight endogenous genes in rice (Figure 3A,B), six of which were experimentally validated due to remarkable transcript reduction in *miR162a-OE* plants (Figure 3D). Little is known about the functions of these genes except *OsDCL1* encoding a key enzyme for miRNA biosynthesis in rice. Strong silencing of *OsDCL1* by over 90% resulted in growth arrest at the seedling stage [49]. Surprisingly, the *OsDCL1* expression was suppressed by approximately 35 and 30% upon overexpression of osa-miR162a or osa-miR162-m1, respectively (Figure 3D). Of particular note, *OsDCL1* was not considered as a target gene of osa-miR162-m1 by WMD (Figure 3A), as there are six mismatches between them (Figure S3). We have previously characterized a large number of plant artificial miRNAs (amiRNAs) and found that effective plant amiRNAs often must contain less than two mismatches to the target site [47]. Therefore, the silencing of *OsDCL1* by osa-miR162-m1 was exceptionally interesting. However, since overexpression of osa-miR162-m1 caused no developmental abnormality (Figure 4), *OsDCL1* is unlikely to be the gene giving rise to the root or grain phenotype in transgenic rice plants overexpressing osa-miR162a. It warrants future studies to clarify which of the remaining genes is responsible for the observed root or grain defect.

In summary, this work reported the first cross-kingdom RNAi mechanism in rice-BPH interactions and revealed osa-miR162a and *NlTOR* as the mobile rice miRNA and the BPH target gene, respectively, in this process. We also translated this finding into molecular breeding of BPH resistance in rice by overexpressing osa-miR162a, and further devised a widely useful strategy to retain the beneficial cross-kingdom RNAi effects but minimize undesirable perturbation on host gene expression for overexpressed plant miRNAs.

## 4. Materials and Methods

### 4.1. Plant and Insect Materials

The rice variety ZH11 was used as wild-type plants. Rice plants were routinely grown in a greenhouse with a cycle of 14 h light (200 μmol/m^2^/s) at 28 °C and 10 h dark at 25 °C. To compare the phenotypes of rice young seedlings, seeds harvested from *miR162a-OE* or *miR162-m1-OE* plants at the same time were surface-sterilized and germinated on 1/2 Murashige & Skoog agar medium in a growth chamber under the conditions described above. A laboratory strain of brown planthopper was obtained from the Guangdong Academy of Agricultural Sciences, China.

### 4.2. Molecular Cloning and Transforming Rice Plants

Routine molecular cloning procedures were followed for plasmid construction. To generate the osa-miR162a-m1 precursor, the osa-miR162a precursor was subjected to PCR-based site-directed mutagenesis. The precursor of osa-miR162a or osa-miR162a-m1 was inserted into the binary vector pCAMBIA1300 between *Avr*II and *Sac*I restriction sites for overexpression. The binary vectors were transformed into *Agrobacterium tumefaciens* strain EHA105 cells through electroporation. Agrobacteria containing the pCAMBIA1300-pre-miR162a or pCAMBIA1300-pre-miR162a-m1 plasmid were used for transforming rice callus. Transformed rice callus cells were selected by hygromycin resistance and regenerated into whole plants according to a standard protocol [50].

### 4.3. Feeding BPH with Osa-miR162a Mimics

For rearing on artificial diets, glass cylinders of 9.0 cm in length and 2.0 cm in diameter were used as feeding chambers. The artificial diet was coated with synthetic osa-miR162a mimics (RIBBIO, Guangzhou, China) to a final concentration of 100 ng/μL, and then placed between two layers of stretched Parafilm that were located at two open ends of the chamber. The artificial diets were renewed every day. The chambers were covered with a piece of black cotton cloth, and the two ends with artificial diets were exposed to light. The insects could eat by puncturing the inner Parafilm layer of the food pouch. Ten female adults of the first day were placed in each chamber, and sets of 100 individuals were considered as a biological replicate in each group. Three replicates were performed. The midgut, fat body, hemolymph, and other tissues from the female adults after 24, 48, and 72 h feeding were dissected in insect saline containing 0.75% NaCl and stored at −80 °C for further use.

### 4.4. Injecting BPH with Osa-miR162a Mimics

Female adults of the first day were used for the injection experiment. The osa-miR162a mimics of 50 ng (1 ng/nL) were injected into the side of the abdomen of the nymph using a NARISHIGE IM-31 microinjector (Nikon, Tokyo, Japan). A control was performed using an equivalent amount of negative control miRNA mimics. Sets of 100 individuals were considered as a biological replicate in each group. Ten individuals were set for a group for 24, 48, and 72 h sampling. Three replicates were performed.

### 4.5. Bioassay of the Fecundity

In the experiments evaluating the fecundity of BPH, each newly emerged female adult after injection with osa-miR162a mimics was transferred to a fresh rice plant, and single paired with an untreated male adult. Controls were performed in which an equivalent volume of negative control miRNA mimics was injected into the females. The treatment group and control group each included 20 pairs of adults. After the eggs were hatched, the newly hatched nymphs were counted and removed every day. Then, rice was dissected and counted under the microscope 15 days later. We summed the numbers of hatched nymphs and unhatched eggs as the total number of eggs. The hatching rate was calculated by dividing the number of hatched nymphs against the total number of eggs. Eggs with no sign of development or completely developed eggs containing no nymphs were considered as unhatched eggs.

### 4.6. RT-qPCR or Stem-Loop RT-qPCR

Total RNA was isolated from BPH female adults or rice using the TRIzol reagent (Invitrogen, CA, USA). Regular or stem-loop RT-qPCR was performed as previously described [51]. Briefly, total RNA was converted into the first-strand cDNA with stem-loop RT primers for osa-miR162a or osa-miR162a-m1 or with an oligo dT primer for other genes using the PrimeScript RT reagent Kit plus genomic DNA Eraser (TaKaRa, Tokyo, Japan). The qPCR was performed in a LightCycler 96 Instrument (Roche, Indianapolis, IN, USA) using the SYBR^®^ Premix Ex TaqTM Kit (TaKaRa, Tokyo, Japan). The relative miRNA or transcript abundance was normalized to the housekeeping gene *Os18s* (*18S rRNA*, *Os09g00999*) in rice or *NlGAPDH* (*Glyceraldehyde-3-phosphate dehydrogenase*) in BPH. The qPCR primers used in this study are listed in Supplementary Table S1.

### 4.7. Feeding BPH with miR162a-OE or miR162a-m1-OE Plants

Five pregnant female adults feeding on ZH11 plants were transferred to fresh ZH11, *miR162a-OE*, or *miR162a-m1-OE* plants. After continuous feeding from parents to offspring adults, the midgut and ovary tissues from 10 pregnant female adults were dissected in insect saline containing 0.75% NaCl. Newly hatched BPH nymphs were fed with *miR162a-OE* or *miR162a-m1-OE* plants. As they grew up, a pair of female and male adults were mated, and the fecundity was analyzed as described above.

### 4.8. BPH Host Choice Test

Eight four-leaf-stage young seedlings of ZH11, *miR162a-OE* or *miR162a-m1-OE* were grown in the same plastic bucket (20 cm in length, 15 cm in width, and 15 cm in height). About 300 BPH nymphs of the third instar were released in the bucket and allowed to choose a host plant. The insects settled on each plant were counted at 48 h after infestation. The survival rate of rice seedlings was determined at the indicated time. Two biological replicates were conducted.

### 4.9. Bioinformatic Prediction of miRNA Target Genes

The target sites of osa-miR162a or osa-miR162a-m1 in *NlTOR* were predicted using the algorithms RNAhybrid (https://bibiserv.cebitec.uni-bielefeld.de/rnahybrid, accessed on 19 November 2021) [52] and miRanda (http://cbio.mskcc.org/miRNA2003/miranda.html, accessed on 19 November 2021) [53]. To predict target genes of osa-miR162a or osa-miR162a-m1 in rice, the WMD was used (http://wmd3.weigelworld.org/, accessed on 19 November 2021) [54]. In the target search program interface of WMD, the 21-nt osa-miR162a or osa-miR162a-m1 sequence was inputted and searched against the database *Oryza sativa* cDNA v6.1, which has been integrated into the program, with the base mismatch setting as five or less.

### 4.10. Statistical Analysis

All the standard statistical analyses were conducted using GraphPad Prism 8.0 software. For multiple comparisons, one-way of variance (ANOVA) with post hoc Tukey tests were used. For two-sample unpaired comparisons, Student’s *t*-tests were used.

## 5. Patents

The data of the current research have applied for a China invention patent (application number: ZL202110873616X).

## Data Availability

All relevant data can be found within the manuscript or its Supplementary Materials on the journal website.

## Supplementary Materials

The following are available online at https://www.mdpi.com/article/10.3390/ijms222312652/s1.
