# Supplementary material for: Overexpression of an Osa-miR162a Derivative in Rice Confers Cross-Kingdom RNA Interference-Mediated Brown Planthopper Resistance without Perturbing Host Development
Source: Int J Mol Sci. 2021 Nov 23;22(23):12652. doi: 10.3390/ijms222312652 (PMC8657652; doi:10.3390/ijms222312652)
Supplement: Supplementary file 1 [file ijms-22-12652-s001.zip › ijms-1449369-supplementary.pdf]

Supplementary Materials

Overexpression of an osa-miR162a derivative in rice confers cross-kingdom RNA interference-mediated brown planthopper resistance without perturbing host development

Wenzhong Shen[1], Shanni Cao[1], Jinghui Liu[1], Wenqing Zhang[1], Jie Chen[2]*, Jian-Feng Li[1]*

[1] State Key Laboratory of Biocontrol, Guangdong Provincial Key Laboratory of Plant Resources, School of Life Sciences, Sun Yat-sen University, Guangzhou, Guangdong 510275, China.

[2] Guangdong Provincial Key Laboratory of High Technology for Plant Protection, Plant Protection Research Institute, Guangdong Academy of Agricultural Sciences, Guangzhou, Guangdong 510640, China.

* Corresponding author

Tel: +86 20 39943513

Fax: +86 20 39943513

E-mail:

Jian-Feng Li: lijfeng3@mail.sysu.edu.cn

Wenqing Zhang: lsszwq@mail.sysu.edu.cn

Jie Chen: chenj@gdppri.com

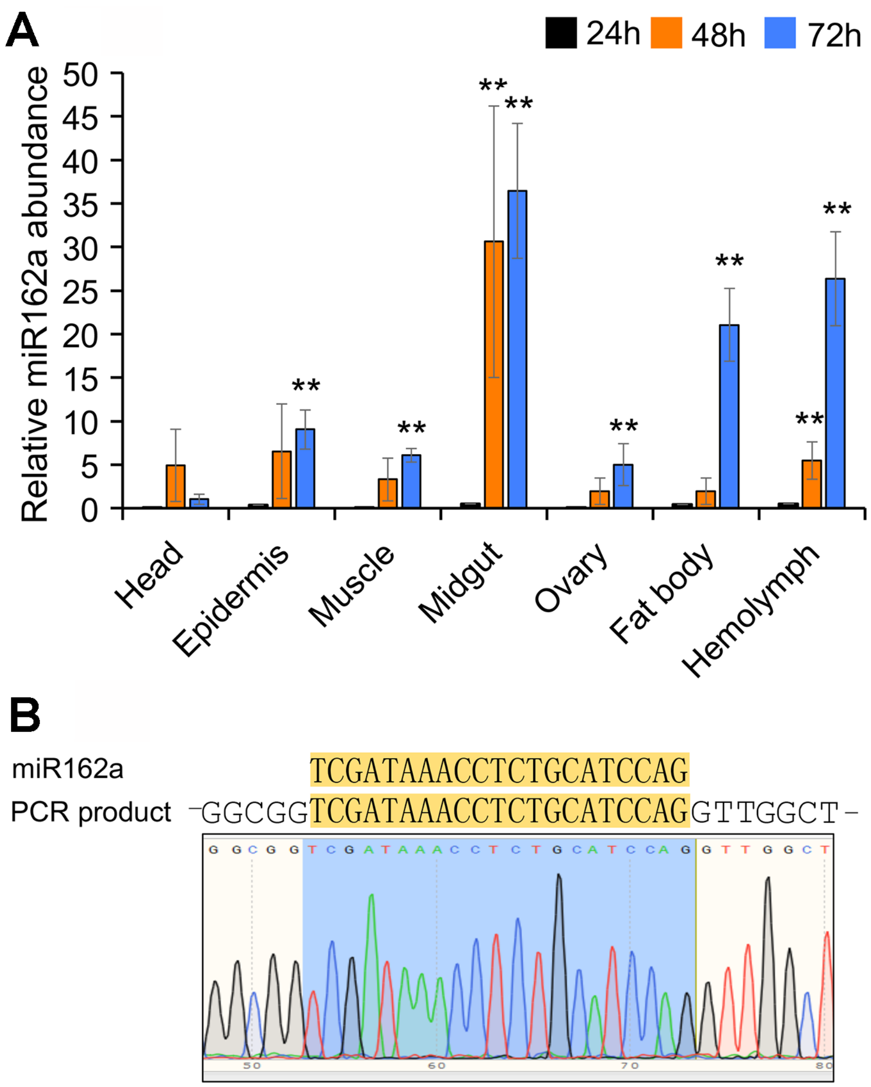

Figure S1. osa-miR162a can be detected in multiple tissues of BPH through artificial diet feeding. (A) Time course analysis of the osa-miR162a presence in various tissues of BPH feeding on osa-miR162a-containing artificial diets. The artificial diets contain synthetic osa-miR162a mimics at a concentration of 100 ng/μl. The abundances of osa-miR162a were determined by stem-loop RT-qPCR and the data were presented as mean ± SD of three biological replicates. The abundance of osa-miR162a in the head after 24 h feeding were normalized as 1. (B) Sanger sequencing of mixed PCR products validates the presence of osa-miR162a inside BPH.

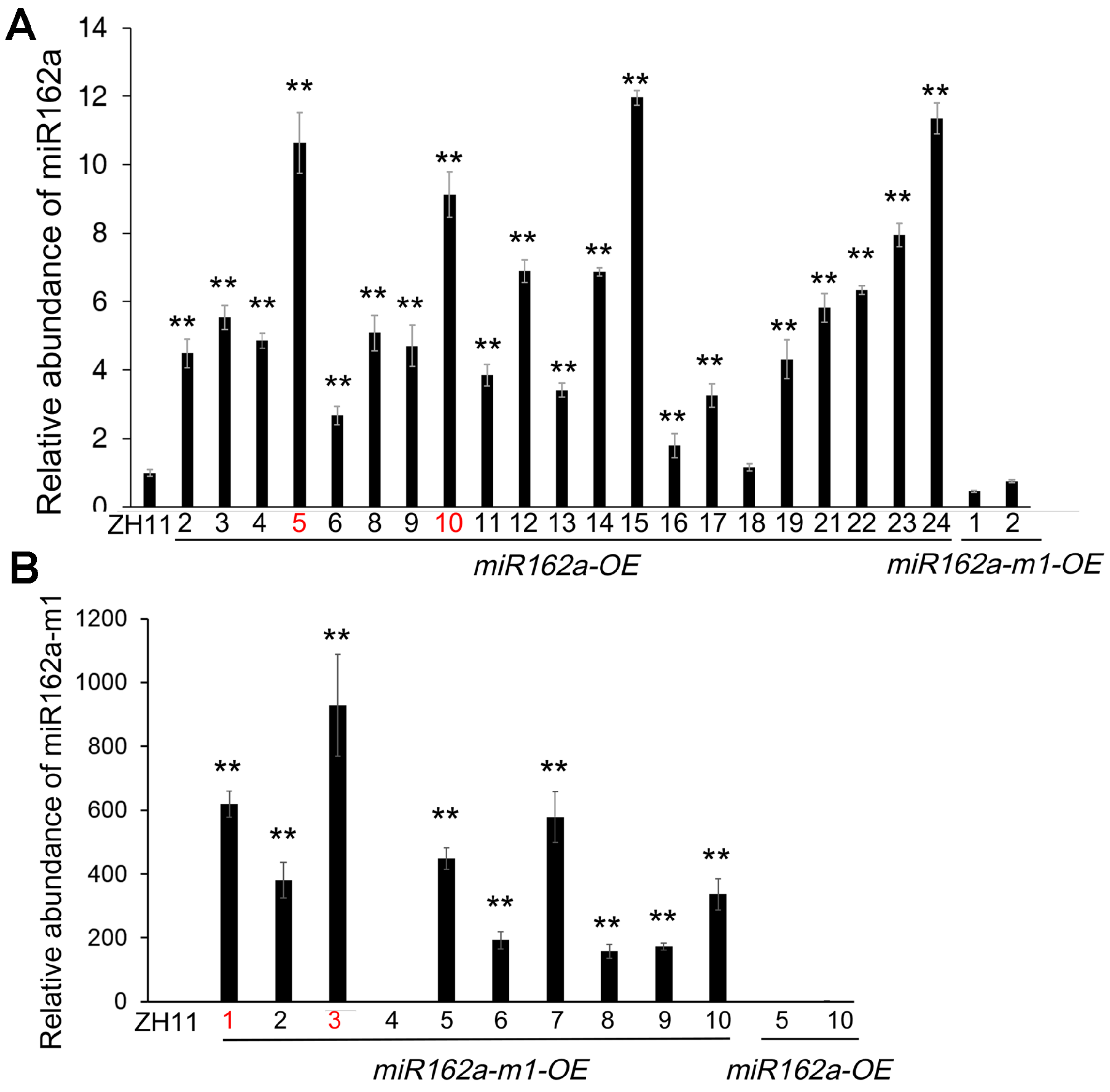

Figure S2. Evaluation of osa-miR162a or osa-miR162a-m1 overproduction in transgenic rice lines. (A) Evaluation of osa-miR162a expression levels in the leaf sheaths of independent T1 *miR162a-OE* lines. The abundances of mature miRNAs were analyzed by stem-loop RT-qPCR. Two *miR162a-m1-OE* lines were assayed as a control to indicate the specificity of qPCR primers used in the experiment. (B) Evaluation of osa-miR162a-m1 expression levels in the leaf sheaths of independent T1 *miR162a-m1-OE* lines. Two *miR162a-OE* lines were assayed as a control to indicate the specificity of qPCR primers used in the experiment. For (A) and (B), the data were presented as mean ± SD of three biological replicates. Two *miRNA-OE* lines used for further analysis are indicated in red.

```
     3' GACCUACGUCUCCAAAU-AGCU 5'    miR162a
        ||||||||||||||||  ||||
5'—AUGAGCUGGAUGCAGAGGUUUUAUCGACGA—3'  OsDCL1
        |||| |||||  ||||  | ||
     3' GACCCACGUCGUCAAAU-AACU 5'    miR162a-m1
```

Figure S3. osa-miR162a and osa-miR162a-m1 have two or six mismatches, respectively, to *OsDCL1*. The modified nucleotides in osa-miR162a-m1 are highlighted in red.

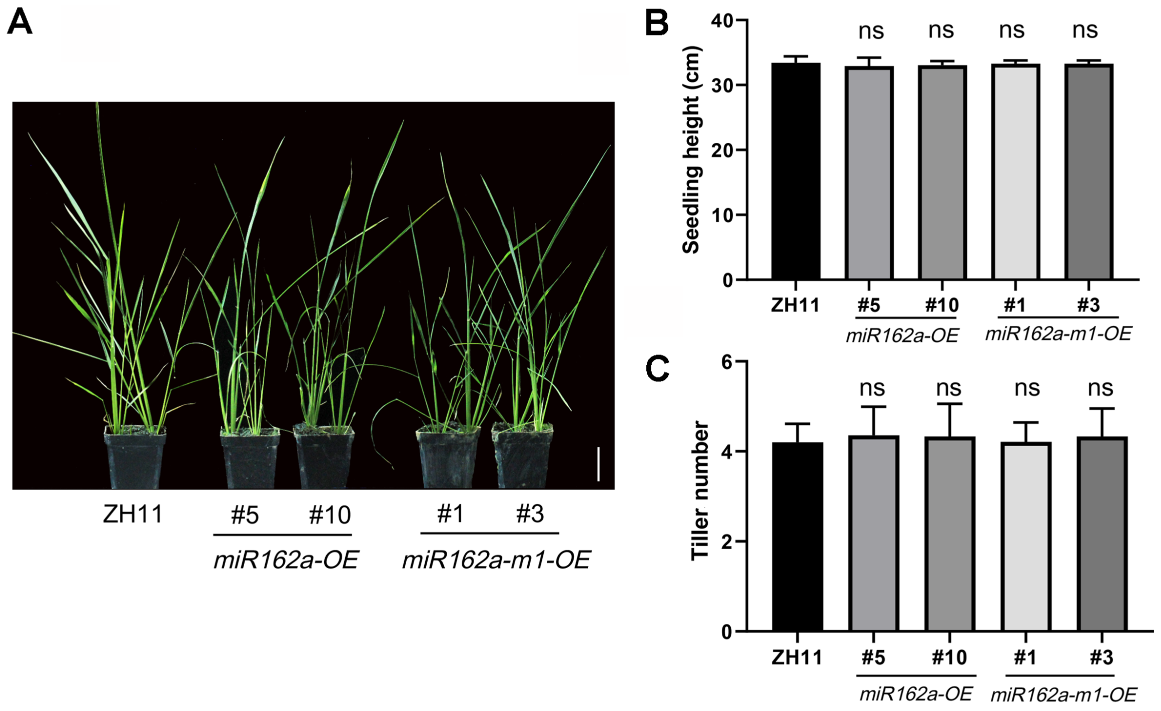

Figure S4. One-month-old *miR162a-OE* and *miR162a-m1-OE* plants exhibit overall wild-type appearances. (A) Representative image for one-month-old *miR162a-OE* and *miR162a-m1-OE* plants. Scale bar = 5 cm. (B, C) Quantification of plant heights (B) or tiller numbers (C) for one-month-old *miR162a-OE* and *miR162a-m1-OE* plants. Twenty randomly selected plants were evaluated and the data are shown as mean ± SD. ** $P < 0.01$ (student's *t*-test).

Table S1. The qPCR primers used in this study

1	osa-miR162a-RT	GTCGTATCCAGTGCAGGGTCCGAGGTATTCGCACTGGATACG ACCTGGAT
2	osa-162a-m1-RT	GTCGTATCCAGTGCAGGGTCCGAGGTATTCGCACTGGATACG ACCTGGGT
3	osa-miR162a-m1-F	GGCCGTCAATAAACTGCTGC
4	osa-miR162a-F	TGCGGTCGATAAACCTCTGC
5	162-universal-R	GTGCAGGGTCCGAGGT
6	miR162a_Target1_F	AGGGTGGGGTATGGACATGA
7	miR162a_Target1_R	CCGTCCTTCTTGAGCCGATT
8	miR162a_Target2_F	AGCACGAAACCTGCATCAGA
9	miR162a_Target2_R	TTCAGATGCCTTCCCAACCC
10	miR162a_Target3_F	GGAGACCACCAGGTTCCAAG
11	miR162a_Target3_R	TCGGCCCAATATCGTCATCC
12	miR162a_Target4_F	GCTGGCCTGTGTTTGTCATC
13	miR162a_Target4_R	GCCGGGATAATCAGGTCCAG
14	miR162a_Target5_F	TGCTGAATATTTCCGCCCCA
15	miR162a_Target5_R	CCAAACCGGACCTGGTATCC
16	miR162a_Target6_F	GCTGAGGACTCGTTCTGTTCA
17	miR162a_Target6_R	AAGAACGGAAAACCTGGGCA
18	miR162a_Target7_F	TATTTTGAGGGCGTCGTGGA
19	miR162a_Target7_R	CGGCCTTGGTCTATCACCTG
20	miR162a_Target8_F	GGCCCGTGGTGTTTCATATT
21	miR162a_Target8_R	CACCTTCTCTAGCTGTTGAACAAA
22	Os18srRNA_F	CTACGTCCCTGCCCTTTGTACA
23	Os18srRNA_R	ACACTTCACCGGACCATTCAA
24	*NlGAPDH*_F	CAAGGTCATCTCCAACGCCT
25	*NlGAPDH*_R	CAGCTTTCCAGAAGGTCCGT
26	*NlTOR*_F	GCTGCTGACCATCCTACTGG
27	*NlTOR*_R	TGTCTCGCGCCTGATAACAA
28	*NlVg*_F	GGCTCCGAGTCAAGTGACAA
29	*NlVg*_R	GTTGGCACCATCGTGGTAGA
30	*NlS6K*_F	TCACATACGTAGCGCCATCC
31	*NlS6K*_R	GTCCATCATTTCGTCGTGCG
